# Pre-analytical stability of blood samples transported by drone versus ground: a pilot study

**DOI:** 10.1016/j.plabm.2026.e00530

**Published:** 2026-04-09

**Authors:** Marie Brionne, Laure Peyro-Saint-Paul, Jean-Jacques Dutheil, Denis Vivien, Hélène Legros, Simon Le Hello, Rémy Morello

**Affiliations:** a Hematology Laboratory, Caen Normandie University Hospital, France; b Department of Clinical Research, Caen Normandie University Hospital, CHU, Caen, France; c Center for Biological Ressources (CRB InnovaBIO), Caen-Normandie University Hospital, CHU, Caen, France; d Normandie Univ, UNICAEN, UNIROUEN, CHU de Caen Normandie, Department of Infectious Agents, Infection Prevention and Control Service, INSERM U1311 DYNAMICURE, 14000, CAEN, France; e Caen Normandie University Hospital, Biostatistics and Clinical Research Unit, 14000, CAEN, France

**Keywords:** Blood samples, Drone transport, Biochemical, Hematological parameters, Hemostasis, Laboratory quality

## Abstract

**Objectives:**

Pre-analytical conditions are critical to ensure the reliability of laboratory results, as emphasized by ISO 15189 standards. Drone transport has emerged as a promising alternative to conventional logistics, but its impact on sample integrity remains insufficiently characterized. This pilot study aimed to assess the pre-analytical stability of blood samples transported by drone versus ground transport.

**Methods:**

In this prospective study, 30 healthy volunteers were included. Six blood tubes per participant were collected simultaneously and assigned to ground or drone transport (20 min). A panel of 23 biochemical, hematological, and hemostatic parameters was analyzed. Agreement between transport modalities was assessed using paired comparisons, coefficients of variation, intraclass correlation coefficients (ICC), Pearson correlation, and Bland–Altman analysis.

**Results:**

No clinically meaningful differences were observed between transport modalities. Mean values and variability were comparable across parameters. Most analytes showed excellent agreement, with ICC and Pearson correlation coefficients >0.90. Although ALT and LDH showed statistically significant differences (p = 0.039), these were small and clinically negligible. Bland–Altman analysis confirmed minimal bias for ALT (−0.63 U/L), whereas LDH exhibited wider limits of agreement, suggesting increased sensitivity to transport-related factors (−9.7 U/L). No hemolysis, temperature deviation, or safety incidents were observed.

**Conclusions:**

Drone transport ensures robust pre-analytical stability of blood samples across a wide range of laboratory parameters. However, analyte-specific variability, particularly for LDH, highlights the need for targeted validation. These findings support the integration of drone-based logistics into laboratory workflows, while emphasizing the importance of analyte-dependent evaluation.

## Highlights

• Drone transport preserves sample integrity vs ground methods.
• Strong analytical agreement across most biomarkers.
• LDH shows variability, requiring analyte-specific validation.

## Graphical abstract

**Figure:**
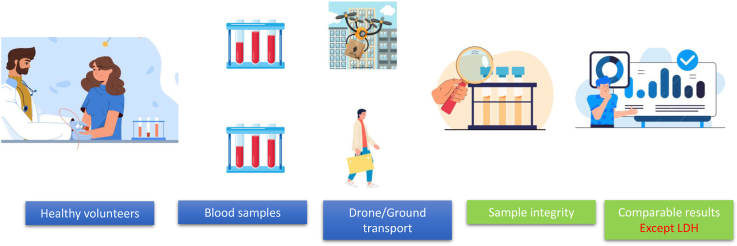

## Abbreviations:

ISO –: International Organization for Standardization
CV –: Coefficient of Variation
ICC –: Intraclass Correlation Coefficient
ESR –: Erythrocyte Sedimentation Rate
Na^+^ –: Sodium
K^+^ –: Potassium
Cl^-^ –: Chloride
HCO_3_^-^ –: Bicarbonate
TP –: Total Proteins
Ca^2+^ –: Calcium
Cr –: Creatinine
Urea –: Urea
Alb –: Albumin
Bili –: Bilirubin
ALP –: Alkaline Phosphatase
CRP –: C-reactive Protein
GGT –: Gamma-Glutamyl Transferase
AST –: Aspartate Aminotransferase
ALT –: Alanine Aminotransferase
LDH –: Lactate Dehydrogenase
RBC –: Red Blood Cells
Hb –: Hemoglobin
MCV –: Mean Corpuscular Volume
PLT –: Platelets
WBC –: White Blood Cells
Neu –: Neutrophils
Lym –: Lymphocytes
PT –: Prothrombin Time
aPTT –: Activated Partial Thromboplastin Time
Fib –: Fibrinogen
DD –: D-dimers
R [2] –: Coefficient of Determination
p-value –: Probability Value
GPS –: Global Positioning System

## 1 Introduction

The transport of biological samples is a crucial pre-analytical step within healthcare facilities. It must adhere to strict quality requirements, such as those defined by ISO 15189 [12], to ensure: (i) compliance with appropriate timeframes based on the nature of the requested tests and specific medical disciplines, (ii) maintenance of the proper temperature during collection and transport, along with the use of recommended stabilizing agents to preserve sample integrity, (iii) safety of transport for both the samples and personnel, including carriers, the general public, and laboratory staff.

These conditions are essential to prevent pre-analytical errors and ensure reliable biological results [2].

Recently, drone-based aerial transport has emerged as an innovative solution across various sectors, including healthcare [[3], [4], [5]]. This technology could provide hospitals and laboratories with rapid, agile, and reliable transportation while reducing environmental impact and promoting resource sharing. Potential advantages of drone transport include: (i) significant reduction in transport time, with direct flight paths enabling up to five-fold faster delivery compared to traditional road transport [1], (ii) optimized workload on laboratory platforms through more regular sample arrivals, (iii) up to 90% reduction in carbon footprint compared to conventional transport [2], (iv) decreased logistical costs, with estimated savings of 25% relative to road transport [2].

Previous studies have evaluated the feasibility of drone transport for chemistry [3], microbiology [4], and hematology [5] samples. For example, Amukele et al. assessed the quality of blood products after a 22-min drone flight at 100 m altitude, demonstrating sample stability [5].

The DBT (Drone Biological Transport) research project (ID RCB: 2022 A02548 35; CHU: 22 0238) was a pilot study conducted at CHU Caen (France), in collaboration with Delivrone®, to assess the analytical comparability of a wide range of biological parameters following drone versus ground (pedestrian) transports. The study focused on analytical quality, personnel safety, transport time, and temperature control (15–25 °C), using a panel of 23 laboratory tests on samples from 30 healthy volunteers.

## 2 Materials and methods

### 2.1 Population

Thirty healthy volunteers participated in the study after receiving detailed information. Samples were anonymized upon collection. Informed non-opposition consent was obtained. Volunteers received €20 in cultural gift vouchers and were registered on the Biomedical Research Volunteers (VRB) platform.

### 2.2 Study site

The study was conducted at the Caen Normandie University Hospital, France.

### 2.3 Sample collection and transport

Each participant provided six blood tubes simultaneously: 2 citrate tubes, 2 lithium heparin gel tubes, and 2 EDTA tubes (total 30 mL). Samples were transported either on ground transport or by drone (3 tubes per modality). Ground transport was performed on foot using insulated carriers, with a duration matched to drone flight time (20 min).

Drone Transport: Samples were placed in a secure, insulated bag equipped with a temperature sensor and GPS tracker (TRIVIA model) (Photo). Drone flights used the CK23 model (CAVOK), with departure and return from a designated site complying with aviation safety regulations. Flights were performed only under favorable weather conditions (wind ≤60 km/h, no precipitation, sufficient visibility).

### 2.4 Analytical methods

Upon receipt, samples were centrifuged and analyzed using automated laboratory analyzers. Parameters included:

Biochemistry: Sodium, potassium, chloride, bicarbonate, total proteins, calcium, creatinine, urea, albumin, bilirubin, ALP, CRP, GGT, AST, ALT, LDH.

Hematology: RBC, hemoglobin, MCV, platelets, WBC, neutrophils, lymphocytes, ESR.

Hemostasis: PT, aPTT, fibrinogen, D-dimers.

Samples were stored for 48 h according to laboratory procedures for quality control, then disposed of via the Infectious Healthcare Waste system.

### 2.5 Outcomes

Primary: Analytical integrity of blood samples between drone and ground transport.

Secondary: Personnel safety, sample integrity (tube condition and seal), and temperature control (15–25 °C).

### 2.6 Statistical analysis

Data were analyzed using paired statistical methods. Agreement between transport modalities was assessed using:

- Coefficients of variation (CV)
- Intraclass correlation coefficients (ICC)
- Pearson correlation coefficients
- Bland–Altman plots (ALT and LDH)

A p-value <0.05 was considered statistically significant. All analysis were performed with IBM-SPSS 23.0.

## 3 Results

### 3.1 Primary outcome

#### 3.1.1 Global findings

On June 27, 2023, a total of 180 blood samples were analyzed. The weather conditions on the day of the study were considered suitable for flight. Transport conditions were fully compliant with protocol requirements. No sample degradation, hemolysis, leakage, or safety incident was observed. Temperature remained within the predefined range (15–25 °C) throughout transport.

#### 3.1.2 Interpretation of the large comparative Table 1

.

**Table 1:** Blood parameters table.

Parameter	Ground transport (Mean ± SD)	CV %	Drone Transport (Mean ± SD)	CV %	p-value	ICC	ICC p	Pearson r	Pearson p
Na (mmol/L)	139.7 ± 1.4	1.0	139.9 ± 1.2	0.9	0.283	0.862	<0.001	0.871	<0.001
K (mmol/L)	3.96 ± 0.26	6.6	3.96 ± 0.19	4.8	0.904	0.784	<0.001	0.822	<0.001
Cl (mmol/L)	105.2 ± 1.64	1.6	105.3 ± 1.80	1.7	0.415	0.926	<0.001	0.930	<0.001
HCO3^-^ (mmol/L)	24.57 ± 1.99	8.1	24.80 ± 1.77	7.1	0.305	0.790	<0.001	0.795	<0.001
Proteins (g/L)	74.20 ± 3.63	4.9	74.27 ± 3.48	4.7	0.791	0.927	<0.001	0.928	<0.001
Calcium (mmol/L)	2.48 ± 0.12	4.8	2.46 ± 0.11	4.5	0.236	0.816	<0.001	0.818	<0.001
Creatinine (μmol/L)	69.90 ± 11.30	16.2	70.30 ± 11.48	16.3	0.130	0.992	<0.001	0.993	<0.001
Urea (mmol/L)	4.47 ± 0.72	16.1	4.46 ± 0.72	16.1	0.599	0.990	<0.001	0.990	<0.001
Albumin (g/L)	45.10 ± 2.80	6.2	45.0 ± 2.68	6.0	0.476	0.962	<0.001	0.962	<0.001
Bilirubin (μmol/L)	12.27 ± 6.65	54.8	12.37 ± 6.72	53.8	0.326	0.997	<0.001	0.997	<0.001
ALP (UI/L)	72.80 ± 21.61	29.7	72.83 ± 21.24	29.2	0.623	0.998	<0.001	0.996	<0.001
CRP (mg/L)	2.13 ± 2.67	125.4	2.10 ± 2.51	119.5	0.316	0.998	<0.001	1.000	<0.001
GGT (UI/L)	29.40 ± 19.63	66.8	29.57 ± 20.07	67.9	0.444	0.998	<0.001	0.998	<0.001
AST (UI/L)	27.33 ± 8.60	31.5	26.87 ± 8.20	30.5	0.129	0.981	<0.001	0.982	<0.001
ALT (UI/L)	28.87 ± 8.20	28.4	28.63 ± 16.24	56.7	0.039	0.995	<0.001	0.995	<0.001
LDH (UI/L)	282.27 ± 47.39	16.8	272.57 ± 51.90	19.0	0.039	0.595	<0.001	0.597	<0.001
RBC (T/L)	4.71 ± 0.41	8.7	4.70 ± 0.42	8.9	0.188	0.991	<0.001	0.991	<0.001
Hb (g/dL)	13.85 ± 1.15	8.3	15.52 ± 1.17	7.5	0.256	0.989	<0.001	0.989	<0.001
MCV (fL)	88.88 ± 3.88	4.4	88.6 ± 3.64	4.1	0.079	0.988	<0.001	0.988	<0.001
Platelets (G/L)	264.33 ± 62.64	23.7	265.23 ± 60.92	23.0	0.552	0.991	<0.001	0.992	<0.001
WBC (G/L)	6.31 ± 1.40	22.2	6.41 ± 1.47	22.9	0.062	0.980	<0.001	0.981	<0.001
NEUT (G/L)	3.52 ± 1.19	33.8	3.56 ± 1.25	35.1	0.075	0.994	<0.001	0.995	<0.001
LYMPH (G/L)	2.05 ± 0.44	21.5	2.09 ± 0.45	21.5	0.185	0.955	<0.0001	0.955	<0.001
ESR (mm/h)	4.43 ± 3.07	69.3	4.97 ± 3.10	62.4	0.226	0.708	<0.001	0.708	<0.001
PT (%)	115.03 ± 14.22	12.4	113.80 ± 13.25	11.6	0.071	0.966	<0.001	0.968	<0.001
aPTTT (s)	24.57 ± 2.16	8.8	24.80 ± 2.12	8.5	0.070	0.950	<0.001	0.950	<0.001
Fibrinogen (g/L)	2.76 ± 0.56	20.3	2.77 ± 0.55	19.9	0.677	0.998	<0.001	0.988	<0.001
D-Dimers (ng/mL)	307.23 ± 230.66	75.08	305.20 ± 222.95	73.05	0.675	0.993	<0.001	0.994	<0.001

The comprehensive table provides a robust statistical comparison across 23 analytes and allows several key observations:

#### 3.1.3 Absence of systematic bias

Mean values between drone and ground transport are nearly identical for all parameters, indicating no systematic analytical shift induced by drone transport.

#### 3.1.4 Preservation of analytical precision

The coefficients of variation are highly comparable between the two transport methods, demonstrating that drone transport does not introduce additional variability.

#### 3.1.5 Reproducibility

Most ICC values exceed 0.95, reflecting excellent agreement and reproducibility between methods. Similarly, Pearson correlation coefficients are consistently above 0.90, confirming strong linear relationships.

#### 3.1.6 Statistical vs clinical significance

Although ALT and LDH show statistically significant differences (p = 0.039), these differences are small, remain within reference ranges, and show substantial overlap, indicating no clinical impact.

#### 3.1.7 Specific case of LDH

LDH exhibits lower agreement metrics (ICC ≈ 0.60), suggesting greater sensitivity to transport conditions. This finding is consistent with previous reports identifying LDH as a marker sensitive to pre-analytical stress [6,7].

#### 3.1.8 Correlation analyses

**Fig. 1:**
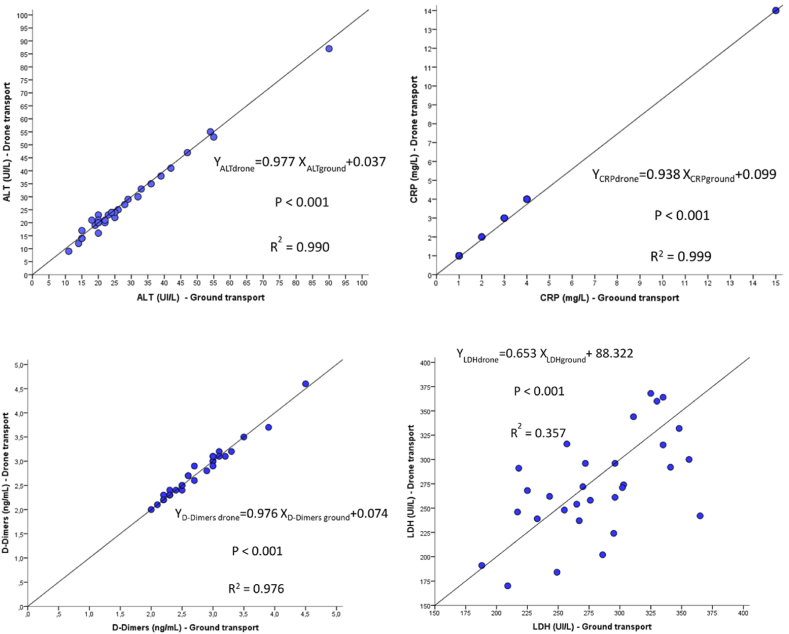
Correlation between biomarker values measured after drone and ground transport. Each point represents one or several samples with identical values.

Strong correlations were illustrated for key analytes (Fig. 1): ALT (R^2^ = 0.990); CRP (R^2^ = 0.999); D-dimers (R^2^ = 0.976).

These findings confirm the robustness of drone transport across a wide analytical range.

On the contrary, LDH shows a low correlation (R^2^ = 0.357) (Fig. 1).

#### 3.1.9 Bland–Altman analyses

**Fig. 2:**
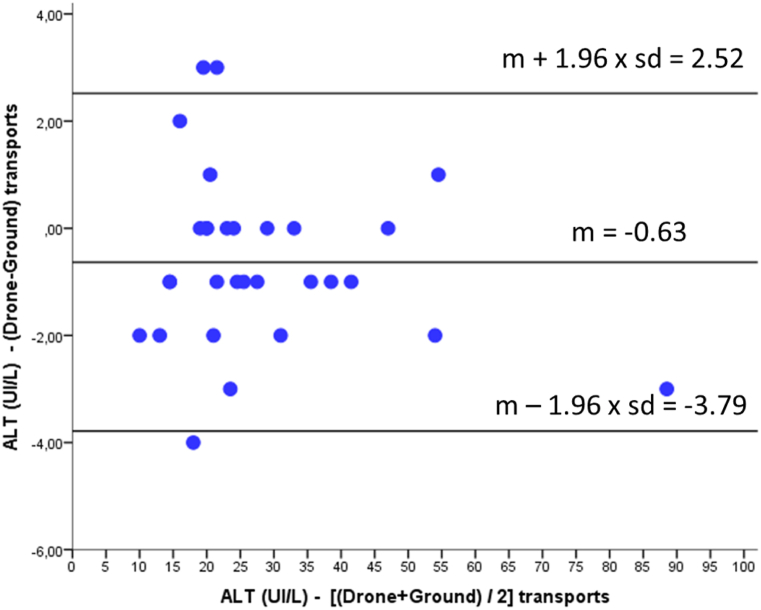
Bland–Altman plot comparing ALT (U/L) measurements obtained after drone versus ground transport. The mean bias was −0.63 U/L, indicating a minimal underestimation of ALT values following drone transport. The 95% limits of agreement ranged from −3.79 to 2.52 U/L.
- LDH: wider limits of agreement (−97.9 to 77.9 U/L); increased variability (Fig. 3).

Bland–Altman analysis revealed important differences between analytes:

- ALT: minimal bias (−0.63 U/L) and narrow limits of agreement; excellent interchangeability (Fig. 2)

**Fig. 3:**
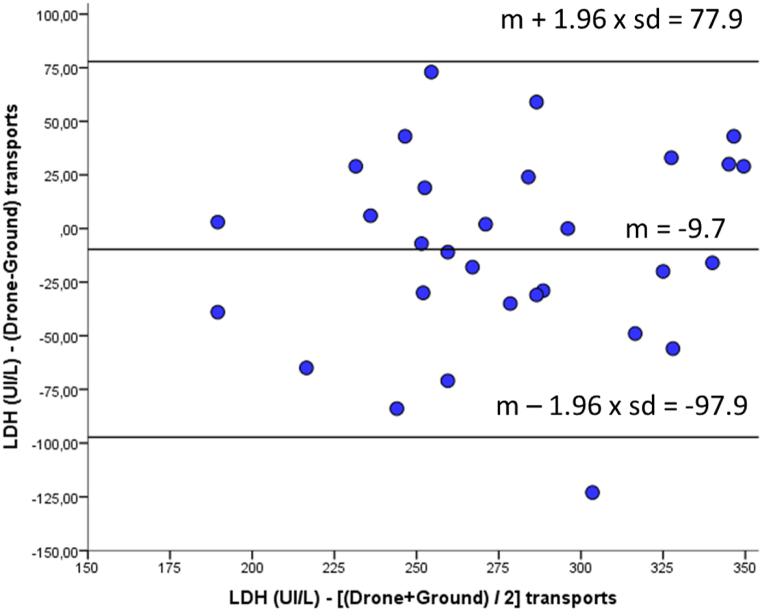
Bland–Altman plot comparing LDH (U/L) measurements obtained after drone versus ground transport. The mean bias was −9.7 U/L, indicating a slight underestimation of LDH values following drone transport. The 95% limits of agreement ranged from −97.9 to 77.9 U/L.

**Photo:**
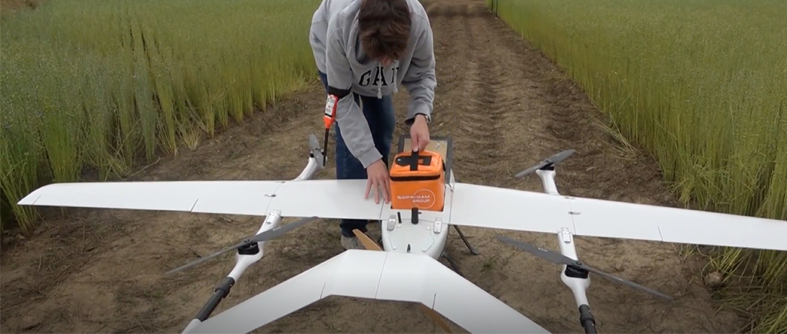
CK23 drone.

These results highlight that correlation alone is insufficient to assess method equivalence.

### 3.2 Secondary outcomes

No safety incidents occurred. All tubes remained intact, with no hemolysis, leakage, or seal loss. Temperature remained stable within 15–25 °C for both transport modalities.

## 4 Discussion

This pilot study demonstrates that drone transport provides robust pre-analytical stability across a wide range of biochemical, hematological, and hemostatic parameters [[3], [4], [5]]. The high level of agreement observed indicates that mechanical constraints associated with drone flight, such as vibration and acceleration, do not significantly impact sample integrity under controlled conditions. This is further supported by the absence of hemolysis and the stability of potassium levels, a sensitive marker of red cell disruption.

In contrast, LDH exhibited lower agreement and wider limits of agreement, suggesting increased sensitivity to transport-related stress. This finding is consistent with previous studies and highlights the importance of analyte-specific validation when implementing alternative transport strategies. Similar findings have been reported by Perlee et al. and Weekx et al., who observed variations in LDH following drone transport [6,7]. In the present study, Bland–Altman analysis revealed substantial variability despite acceptable mean differences, indicating that caution is warranted when interpreting LDH results.

An important methodological strength of this study is the combined use of correlation and agreement analyses. While correlation coefficients confirm linear relationships, Bland–Altman analysis provides a more rigorous assessment of interchangeability. This distinction is critical for clinical interpretation.

From an operational perspective, drone transport offers significant advantages, including reduced delivery time, improved logistics, and lower environmental impact [1,2,8]. These benefits are particularly relevant in emergency settings and remote areas, as demonstrated in studies conducted in Rwanda and India [7,[9], [10], [11]].

## 5 Limitations

This study was conducted in healthy volunteers, allowing for a controlled assessment of pre-analytical variability under standardized conditions. However, the impact of drone transport on samples with pathological values remains to be determined, as analyte concentrations (e.g., CRP) were predominantly within normal ranges.

In addition, the study was performed in a single geographic location (Normandy, France), which may limit generalizability to other environmental conditions. Although previous studies conducted in diverse settings such as Rwanda, India, and equatorial regions have reported similar findings, environmental variability may still influence results.

Finally, the sample size was moderate (n = 30), and larger studies may be required to detect subtle effects, particularly for sensitive analytes.

Future studies should explore longer transport durations, different drone models, varied environmental conditions, and a broader range of biomarkers to further validate these findings.

## 6 Conclusions

Overall, drone transport represents a reliable and scalable alternative for biological sample logistics, with potential applications in both routine and remote healthcare settings. Most analytes demonstrated excellent agreement between transport modalities, supporting the robustness of this approach in pre-analytical conditions. However, analyte-specific variability, particularly for LDH, highlights the need for targeted validation. These findings support the integration of drone technology into routine laboratory workflows.

## CRediT authorship contribution statement

**Marie Brionne:** Conceptualization, Data curation, Formal analysis, Investigation, Writing – original draft, Writing – review & editing. **Laure Peyro-Saint-Paul:** Formal analysis, Validation, Writing – original draft, Writing – review & editing. **Jean-Jacques Dutheil:** Funding acquisition, Project administration, Writing – original draft, Writing – review & editing. **Denis Vivien:** Conceptualization, Supervision. **Hélène Legros:** Conceptualization, Investigation. **Simon Le Hello:** Conceptualization, Investigation. **Rémy Morello:** Conceptualization, Formal analysis, Methodology, Writing – original draft, Writing – review & editing.

## Role of the funding source

This work was supported through a scientific collaboration and co-funding between 10.13039/501100015613CHU de Caen and Deliverone, a drone transport company. Deliverone had no role in the study design, data collection, data analysis, data interpretation, or writing of the manuscript.

## Declaration of competing interest

The authors have nothing to disclose.

## Data Availability

Data will be made available on request.
